# Effects of Cooling Rate during Quenching and Tempering Conditions on Microstructures and Mechanical Properties of Carbon Steel Flange

**DOI:** 10.3390/ma13184186

**Published:** 2020-09-21

**Authors:** Haeju Jo, Moonseok Kang, Geon-Woo Park, Byung-Jun Kim, Chang Yong Choi, Hee Sang Park, Sunmi Shin, Wookjin Lee, Yong-Sik Ahn, Jong Bae Jeon

**Affiliations:** 1 Energy Materials and Components R&D Group, Korea Institute of Industrial Technology, Busan 46938, Korea; cat3871@kitech.re.kr (H.J.); kms523@kitech.re.kr (M.K.); dndi9112@unist.ac.kr (G.-W.P.); jun7741@kitech.re.kr (B.-J.K.); smshin@kitech.re.kr (S.S.); wkjinlee@kitech.re.kr (W.L.); 2 Department of Materials Science and Engineering, Pukyong National University, Busan 48513, Korea; 3 Research Center, Felix Technology Co., Ltd., Busan 46733, Korea; ccyrich@naver.com (C.Y.C.); parkhs@fete.co.kr (H.S.P.)

**Keywords:** flange, F70 steel, heat treatment, multiphase microstructure, mechanical property

## Abstract

This study investigated the mechanical properties of steel in flanges, with the goal of obtaining high strength and high toughness. Quenching was applied alone or in combination with tempering at one of nine combinations of three temperatures *T*_TEM_ and durations *t*_TEM_. Cooling rates at various flange locations during quenching were first estimated using finite element method simulation, and the three locations were selected for mechanical testing in terms of cooling rate. Microstructures of specimens were observed at each condition. Tensile test and hardness test were performed at room temperature, and a Charpy impact test was performed at −46 °C. All specimens had a multiphase microstructure composed of matrix and secondary phases, which decomposed under the various tempering conditions. Decrease in cooling rate (CR) during quenching caused reduction in hardness and strength but did not affect low-temperature toughness significantly. After tempering, hardness and strength were reduced and low-temperature toughness was increased. Microstructures and mechanical properties under the various tempering conditions and CRs during quenching were discussed. This work was based on the properties directly obtained from flanges under industrial processes and is thus expected to be useful for practical applications.

## 1. Introduction

The flange is a component used to interconnect pipelines and must provide a strong and reliable connection. Since pipelines are increasingly being developed for harsh environments such as high pressure, low temperature and corrosive atmospheres, flanges are thus also required to have excellent mechanical properties and reliability [1,2,3,4,5].

The mechanical properties of thick steel plates used for large structures such as flanges are controlled using post-heat treatment [6,7,8,9]. Quenching is used for the purpose of improving strength but it has a detrimental effect on low temperature toughness due to the formation of a hard secondary phase. Thus, tempering has been used to obtain a balance of strength and toughness by optimizing the microstructure [4,10]. In low carbon steels, it is well known that tempering after quenching induces decomposition of as-quenched martensite into tempered martensite and precipitates, static recovery of dislocation and release of residual stress [11,12,13,14]. Martensite after tempering loses strength but gains toughness by the release of embedded carbons, which later form precipitates. Not only tempered martensite itself but also the precipitates affect mechanical properties as they vary by their type, morphology and size [11,12]. Generally appropriate control of various process parameters included tempering temperature *T*_TEM_ and holding time *t*_TEM_ can yield the desired mechanical properties.

However, when a thick steel plate is subjected to a given *T*_TEM_ and *t*_TEM_, different parts of it cool at different rates; this variation causes non-uniformity of microstructures and variation in final mechanical properties [8,9,15,16,17]. After hot-forging of the flange, a post-heat treatment process can also be used to control its mechanical properties, but the combination of high strength and high toughness is difficult to obtain due to the non-uniform microstructure and the complicated effects of the parameters of the tempering process.

When dealing with bulk flanges of large dimensional size and complex structure, it should be taken into consideration that heat treatment would not be uniformly applied to each location of the products. However, most previous studies have tended to use standardized specimens [18,19,20,21,22] or sheet plates [9,16] of certain sizes to which uniform heat treatment can be applied within the specimens, so the results cannot be directly used to understand the bulk flanges. It is rather useful, especially from an industrial point of view, to investigate the effect of heat treatment on flanges by directly obtaining local properties of interest from the flanges under heat treatment processes.

Therefore, this study considered 10-inch-diameter weld neck flanges at three locations selected according to cooling rates (CRs) estimated using finite element method (FEM) simulation. Microstructures were investigated at the corresponding locations on the untempered flange and on flanges that had been subjected to one of nine types of tempering conditions (3 *T*_TEM_ × 3 *t*_TEM_). Specimens were cut from each location of each flange and then subjected to tensile and hardness tests at room temperature and a Charpy impact test at −46 °C. The results were used to determine the correlation between microstructure and mechanical properties under various CRs and tempering conditions.

## 2. Background

Low-carbon steel sheets have various microstructures and therefore various characteristics. Microstructures are distinguished into matrix and secondary phase. According to the morphological characteristics, the matrix is classified as granular bainitic ferrite (GBF), lath bainitic ferrite (LBF), acicular ferrite (AF) and quasi-polygonal ferrite (QPF), and the secondary phases are classified as martensite (M), martensite–austenite constituent (MA), degenerated pearlite/pearlite (DP/P) and carbide (C). 

GBF and LBF are two different morphologies of bainitic structure, based on descriptions in the literature [23,24]. GBF consists of block ferrite and a granular M/A constituent. GBF is transformed during continuous cooling and can transform over a wide range of CRs. For these reasons, GBF tends to form a major structure in low carbon steels that have cooled at various rates [17,25,26,27]. After GBF transformation ends, LBF is formed in the region of retained austenite with rich carbon content, but not rich enough to form M/A constituent [11]. LBF has a parallel lath shape and has relatively higher strength than GBF due to the fine laths and rich carbon content [25,26,27]. 

The transformation temperature ranges of AF and GBF are similar; typically, AF is formed at a faster CR than GBF. AF nucleates within grains in austenite as lath-shaped needles that radiate irregularly in various directions to form basket weaves. These chaotically arranged AF laths impede crack propagation so the AF microstructure produces an excellent combination of strength and toughness [17,25,27]. 

QPF transforms at slow CRs at high temperatures. It has higher dislocation density than polygonal ferrite and has irregular grain boundaries. Secondary phases often form at QPF grain boundaries [25]. Compared to other matrices, OPF has the lowest strength but excellent ductility.

M is formed by cooling austenite at a greater than critical CR, which prevents carbon from spreading. As a result, carbon forms a solid solution and M is an unstable microstructure with high dislocation density. M has a lath phase in low-carbon steel, so it has very high hardness and strength, but M is brittle, so its presence degrades toughness. MA consists of martensite and retained austenite. 

The distribution of martensite in MA depends on the stability of retained austenite and affects the mechanical properties of MA [4,17,28,29,30]. 

P is a eutectoid structure composed of ferrite and cementite with a lamellar structure and forms at high temperatures with slow CR. The mechanical properties of P are between those of martensite and ferrite. DP has almost the same mechanical properties as P. DP is transformed by low carbon concentration or increased CR compared to that of P [31].

C is usually observed on grain boundaries and impurities form and aggregate at the end of phase transformation. C is also observed inside GBF and may be finely precipitated by tempering. The influence of C on the mechanical properties depends on the shape and size [32].

## 3. Materials and Methods 

**Figure 1:**
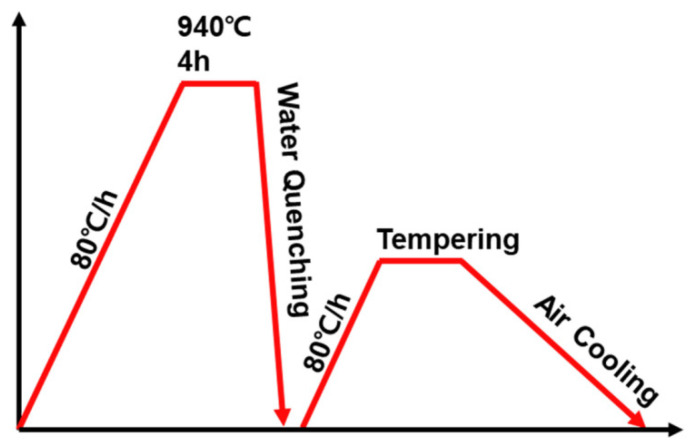
Schematic illustration of post-heat treatment process.

In this study, low-alloy steel (Table 1) was used to make 10-inch-diameter weld neck flanges. The raw steel was heated at 1250 °C for 12 h in a furnace. Hot-forging and punching were performed at 850 to 1250 °C using a hydraulic press. The completed flanges were then subjected to post-heat treatment of the entire flange in a furnace (Figure 1). All flanges were heated at a heating rate of 80 °C/h to 940 °C and held at that temperature for 4 h and quenched in a water bath that had a temperature of 25 to 50 °C. Afterward, flanges were reheated at 80 °C/h to *T*_TEM_ = 450, 530 or 600 °C, held at that temperature for *t*_TEM_ = 1, 3 or 5 h, then air-cooled. Specimens were named according to their tempering conditions (Table 2).

**Figure 2:**
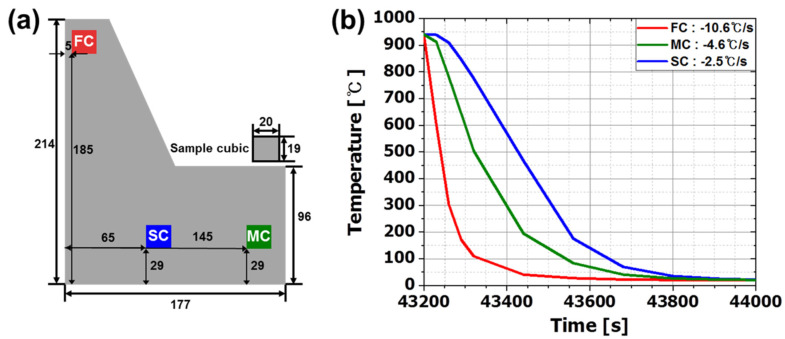
(**a**) Schematic illustration of each specimen location according to CR. Dimensions: mm. (**b**) Cooling curve of each specimen obtained by FEM simulation (Forge NxT 3.0). Colors in (**a**) location and (**b**) cooling curves indicate cooling rates; red, green and blue mean FC (−10.6 °C/s), MC (−4.6 °C/s) and SC (−2.5 °C/s) respectively.

CRs during quenching by location of flange were estimated using FEM simulation (Forge NxT 3.0, Enginsoft, Rome, Italy). Thermal parameters for the heat transfer simulation during annealing and quenching were set as those of a low-alloy steel (C45 steel), provided by the commercial FEM software. Metal to metal contact and metal to fluid quenching were applied to the heat transfer simulation. Three rectangular zones (20 mm × 19 mm) were then selected (Figure 2a): they had fast CR (FC) −10.6 °C/s, middle CR (MC) −4.6 °C/s or slow CR (SC) −2.5 °C/s (Figure 2b). The sample size was defined considering the production of test specimens.

**Figure 3:**
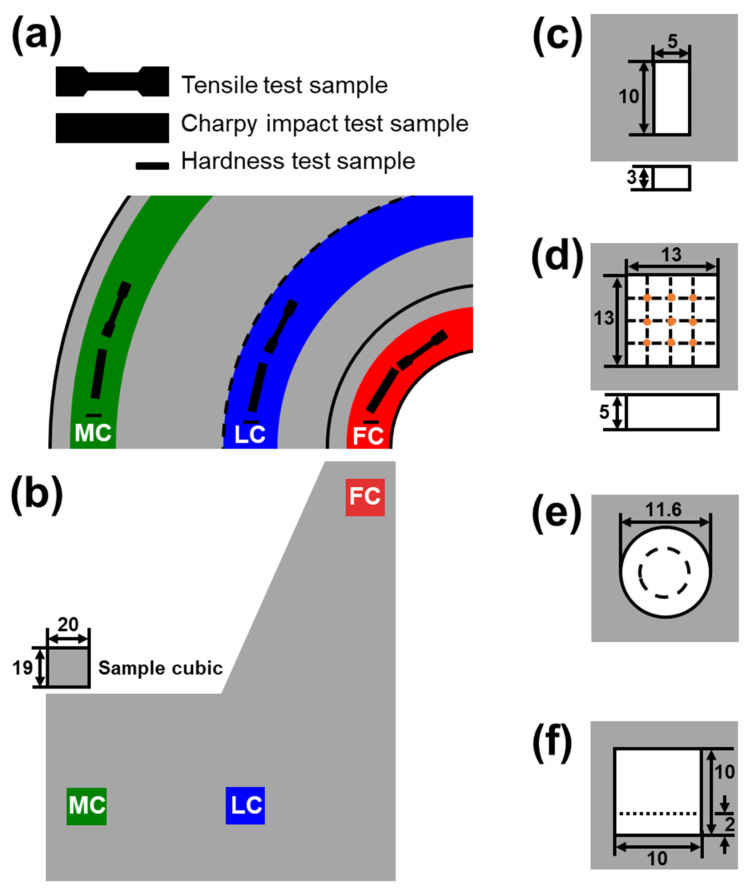
Schematic illustration of testing location in flange for samples. (**a**) Horizontal direction, (**b**–**f**) vertical direction, (**c**) microstructure, (**d**) hardness test (marked as orange), (**e**) tensile test, (**f**) Charpy impact test. Dimensions: mm.

Microstructure observation, hardness test, tensile test and Charpy impact test were conducted using specimens taken at three locations (Figure 3a,b). To observe microstructure, 5 × 10 × 3 mm specimens were obtained along the vertical direction of the flange and then polished physically using SiC paper and diamond paste. Then, they were etched using 4% Nital solution (4% NHO_3_ + ethanol), and then the matrix was observed using an optical microscope (Olympus corporation, GX51F, Okayama, Japan) and the secondary phase was identified using a scanning electron microscope (JEOL, 7200F, Tyoko, Japan) and energy dispersive spectrometer (EDS) (Oxford Instruments, Nanoanalysis, Oxford, England). Using these microphotographs, microstructure types were classified according to their morphological features and their area fractions were calculated. To confirm the presence of MA constituents in the microstructure, the specimens were etched using Klemm’s I solution (50 mL saturated aqueous Na_2_S_2_O_3_ + 1 g K_2_S_2_O_5_) for 40 to 50 s and then observed using an optical microscope. To understand phase evolution during tempering, equilibrium phases were predicted based on themodynamics calculation using Thermocalc with TCFE 9 database (Thermocalc, ver. 2020b).

Hardness tests were performed using a Rockwell hardness (HRC) tester (Hanbando commerce, HBD-DBE SW2259, Seoul, Korea) on specimens with dimensions of 13 × 13 × 5 mm taken along the vertical direction of the flange. Hardness was measured using a C-type scale. Nine measurements were taken from each parts and then the maximum and minimum values were discarded and the average was calculated for the rest. 

Tensile test specimens were round according to sub-size standard of ASTM-E8 and were prepared along the horizontal direction of the flange. The tests were conducted using a tensile testing machine (MTS criterio, model 45, Eden Prairie, MN, USA) and were gauged once at a crosshead speed of 1.5 mm/min at room temperature. 

The Charpy impact test was performed on specimens that had been machined according to ASTM-E23 standard; these samples were taken along the horizontal direction of the flange. Charpy impact tests were conducted once at −46 °C using an impact tester (ZwickRoell, PSW750+TZE, Ulm, Germany).

## 4. Results and Discussion

**Figure 4:**
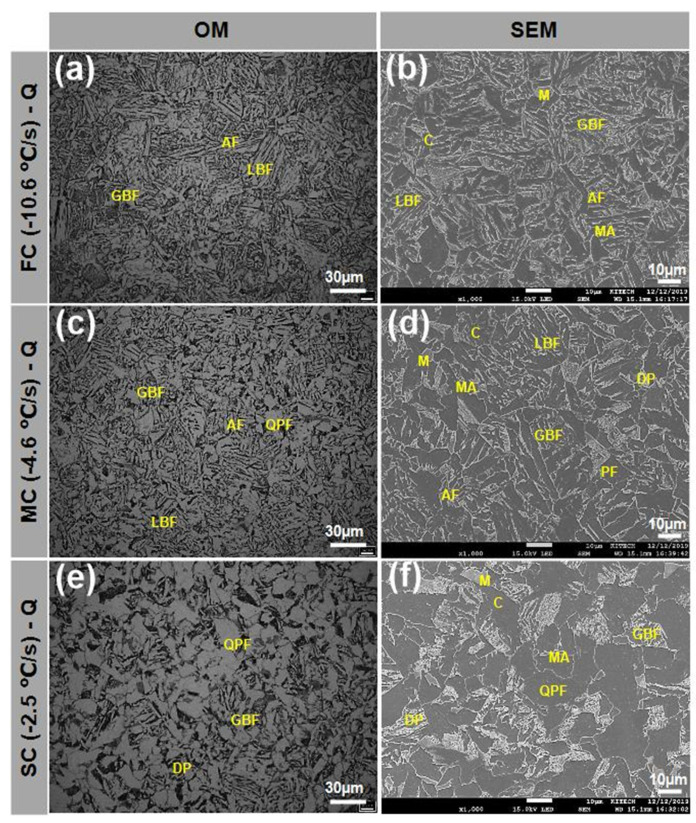
(**a**–**c**) Optical and (**d**–**f**) scanning electron micrographs of Q specimens etched by 4% Nital. (**a**,**d**) FC (−10.6 °C/s), (**b**,**e**) MC (−4.6 °C/s), (**c**,**f**) SC (−2.5 °C/s). (LBF: lath bainitic ferrite, AF: acicular ferrite, GBF: granular bainitic ferrite, QPF: quasi-polygonal ferrite, M: martensite, DP: degenerated pearlite, MA: martensite–austenite constituent).

**Figure 5:**
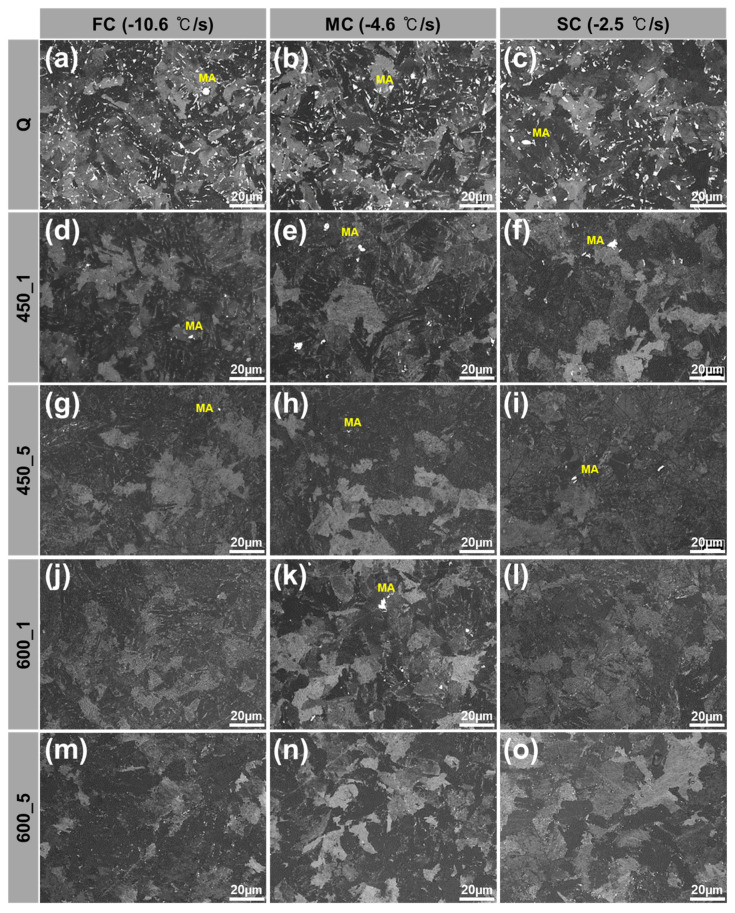
Optical micrographs of microstructure etched by Klemm’s I to distinguish MA (colored as white) according to various CRs and heat treatment conditions: (**a**,**d**,**g**,**j**,**m**) FC (−10.6 °C/s), (**b**,**e**,**h**,**k**,**n**) MC (−4.6 °C/s), (**c**,**f**,**i**,**l**,**o**) SC (−2.5 °C/s), (**a**–**c**) Q, (**d**–**f**) 450_1, (**g**–**i**) 450_5, (**j**–**l**) 600_1, (**m**–**o**) 600_5.

Q-condition specimens had a matrix that included LBF, AF, GBF and QPF and secondary phases M, MA, DP/P and C (Figure 4). These microstructure types were classified by morphological features (Table 3) [23,24,25,33,34]. The area fraction of phases (Figure 5) was measured three times in each and averaged using an image analyzer [35]. C microstructures were smaller than other microstructures and very rare, so C was excluded due to difficulties in identification and measurement of area fraction.

GBF was a major microstructure at all CRs (Table 3). As CR increased, the fractions of LBF, AF and M increased but the fractions of QPF and DP/P decreased. 

In FC-Q (Figure 4a,b), the matrix was mainly composed of GBF (47 ± 4.1% area fraction), followed by AF (16 ± 4.0%) and LBF (13 ± 1.0%), and the secondary phase was mostly M (17 ± 2.0%) with small amounts of DP/P (5 ± 1.5%) and MA (2 ± 0.2%). Because of the fast CR, FC-Q had fine grains; coarse grains were not observed.

In MC-Q (Figure 4c,d), the matrix was mainly composed of GBF (51 ± 4.7% area fraction) but included QPF (6 ± 3.4%), and the secondary phases were M (11 ± 3.1%) and DP/P (10 ± 0.7%); compared to FC-Q, this was a lower area fraction of Am and a higher area fraction of DP/P. The area fraction of MA was still low (2 ± 0.1%), and the grains were coarser than in FC-Q. 

In SC-Q (Figure 4e,f), the matrix was composed of similar area fractions of OPF (39 ± 7.9% area fraction) and GBF (37 ± 5.9%); AF and LBF were not observed. The main secondary phase was DP/P (15 ± 1.4%); M (7 ± 1.3%) was relatively uncommon. The area fraction of MA was the same as in FC-Q and MC-Q. Grains in SC-Q were coarser than in FC-Q and MC-Q, because in SC-Q, the CR was slow so the specimen stayed at a high temperature for a relatively long time.

The area fractions of MA were not affected by CR, unlike the other microstructure types. The effect of CR on the formation of MA has not been established. Previous studies have reported that the area fraction of MA increased [26,35] or decreased [36,37] as CR increased.

The area fraction of MA was significantly reduced after tempering [38,39]. Q specimens (Figure 5a,b,c) had numerous small MA inclusions. In 450_1 specimens, some of the relatively coarse MA remained, but the rest disappeared (Figure 5d,e,f). In 450_5 specimens (Figure 5g,h,i) with increased tempering time and in 600_1 specimens (Figure 5j,k,l) with increased *T*_TEM_, almost all of the MA disappeared. In 600_5 (Figure 5m,n,o) with increases in both *T*_TEM_ and *t*_TEM_, MA was not observed. From the literature [4,10,29,30], the disappearance could be explained by decomposition of MA to ferrite matrix and cementite. Decomposition of MA could be expected to reduce hardness and strength and improve toughness. However, the area fraction of MA was 2% in all specimens before tempering (Table 3) and so should not affect their mechanical properties.

Tempering decomposed the secondary phases. As *T*_TEM_ and *t*_TEM_ increased, cementite became spheroidized and coarsened. Spheroidization of cementite is thermodynamically driven by a decrease in ferrite/cementite interfacial energy. Small cementite belongs to large cementite by Ostwald ripening. These cementites are coarsened and the total surface area to volume ratio is decreased [40,41].

**Figure 6:**
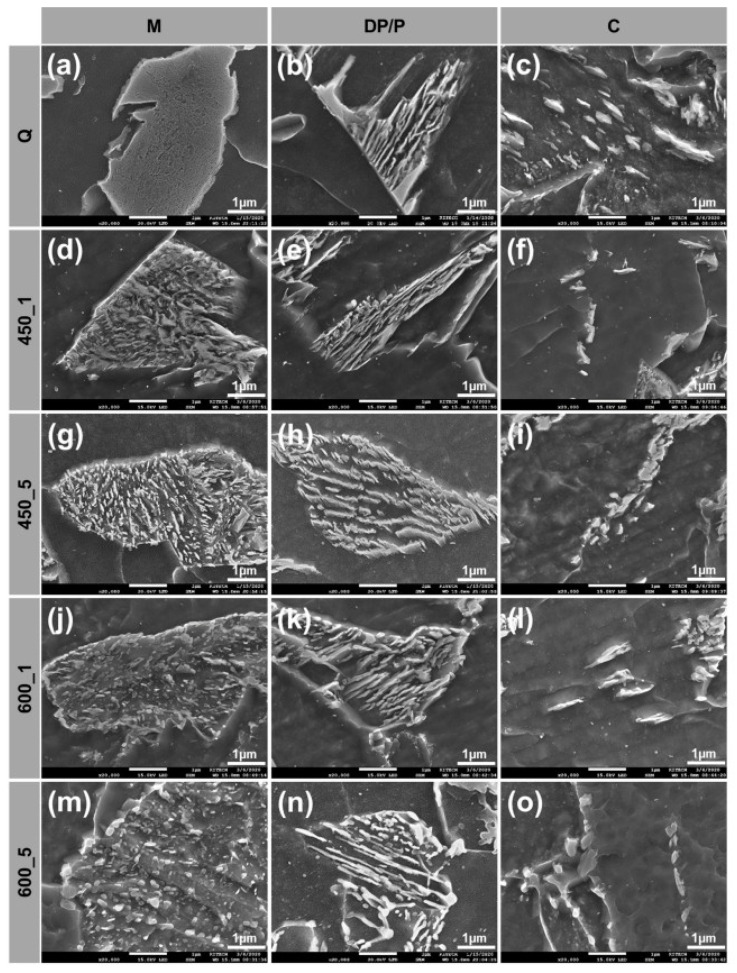
Scanning electron micrographs of (**a**,**d**,**g**,**j**,**m**) martensite, (**b**,**e**,**h**,**k**,**n**) degenerated pearlite/pearlite, (**c**,**f**,**i**,**l**,**o**) cementite according to tempering conditions in the MC (−4.6 °C/s) location: (**a**–**c**) Q, (**d**–**f**) 450_1, (**g**–**h**) 450_5, (**j**–**l**) 600_1, (**m**–**o**) 600_5.

M content decreased as *T*_TEM_ and *t*_TEM_ increased. Comparison to the Q specimen (Figure 6a) indicated that the decomposition of M started in the 450_1 specimen (Figure 6d); pre-existing M broke up into ferrite and cementite, and then they mixed with fine cementite and precipitated after tempering. In the 450_5 specimen (Figure 6g) and the 600_1 specimen (Figure 6j), M was decomposed completely into fine ferrite and tempered martensite. Cementite became coarsened and nearly spherical in the 600_5 specimen (Figure 6m). 

DP/P content was affected by *T*_TEM_ and *t*_TEM_. DP/P content did not differ noticeably between Q (Figure 6b) and 450_1 specimens (Figure 6e). In 450_5 (Figure 6h) and 600_1 specimens (Figure 6k), pre-existing degenerated cementite of DP was spheroidized, and the edges of lamellar cementite of P became rounded. In the 600_5 specimen (Figure 6n), spheroidized cementite was coarsened and non-degenerated lamellar cementite was still present. 

C content was not affected by *T*_TEM_ or *t*_TEM_ and did not differ noticeably between Q specimens (Figure 6c) and 450_1 specimens (Figure 6f). This indicates the occurrence of the decomposition and coarsening of C in 450_5 (Figure 6i) and 600_1 specimens (Figure 6l). In the 600_5 specimen (Figure 6o), C was spheroidized in comparison to the specimens of 450_5 and 600_1. 

**Figure 7:**
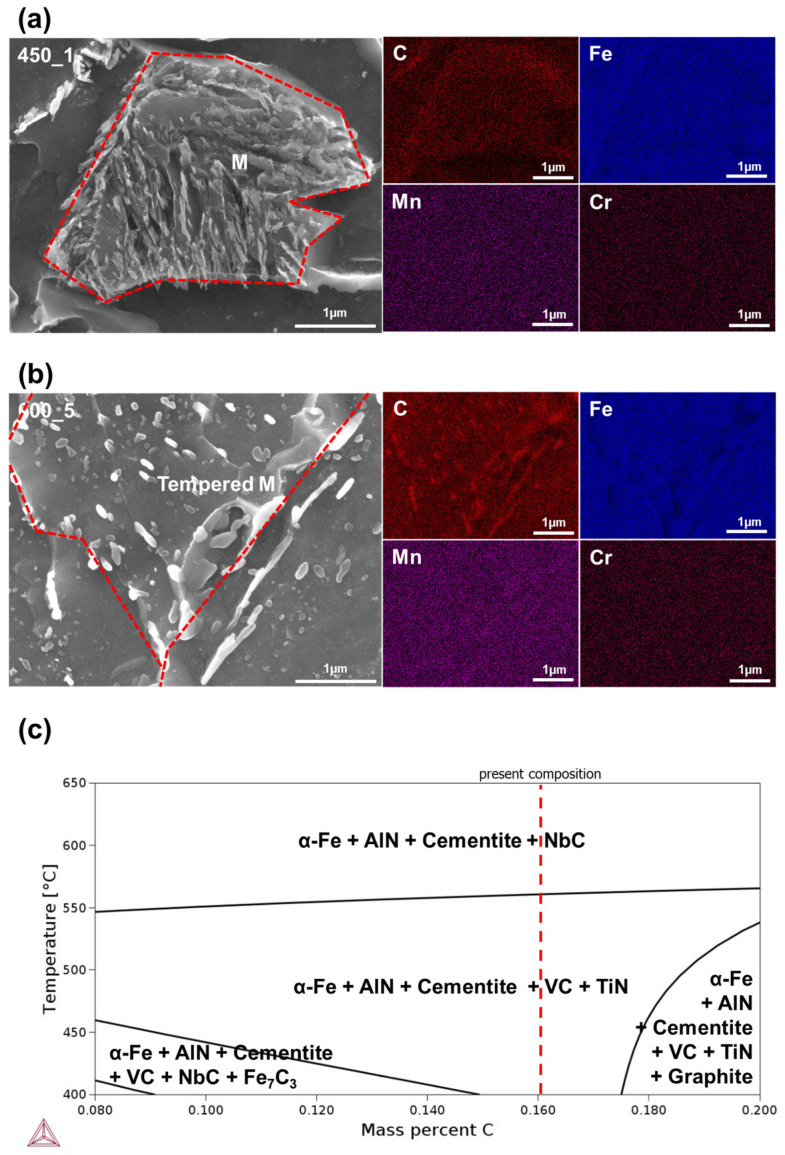
EDS analysis of carbides in decomposed martensite in (**a**) 450_1 and (**b**) 600_5 specimens. (**c**) Calculated equilibrium phase diagram of the present alloy.

It was attempted to identify carbides in decomposed M during tempering and the results are depicted in Figure 7. As can be seen in Figure 7a,b, EDS analyses of carbides in 450_1 and 600_5 specimens clearly revealed that carbide formation was promoted with increasing tempering time and temperature. The stoichiometry of the carbides was close to M_3_C, cementite carbides. These results were well matched with the stable phase predicted by equilibrium thermodynamics calculation, as shown in Figure 7c. Within the present tempering temperatures, cementite was predicted to be stable. This is also in good agreement with previous reports in which decomposition of M into M_3_C cementite carbides was observed [10,42]. 

The mechanical properties of the steels were affected by the decomposition of the secondary phase and the decomposition velocity of each type according to tempering conditions. M decomposed faster than DP/P under the same tempering conditions; this phenomenon has been reported previously [43,44]. M is unstable at high temperatures and has a pronounced tendency to decompose to ferrite and cementite, which are thermodynamically stable microstructures. Furthermore, carbon’s activation energy for diffusion is low, so carbon has a strong tendency to escape the microstructure. In contrast, DP/P is stable and has markedly lower spheroidizing velocity than that of unstable structures [40]. Moreover, tempering drives various phenomena such as the release of residual stress, decline in dislocation density and appearance of fine precipitates. These phenomena combine with the transformation of MA and decomposition of the secondary phase identified to control the mechanical properties of the steels [4,10,19,30,45].

**Figure 8:**
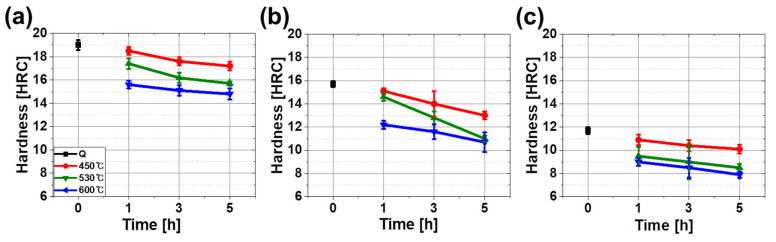
Hardness of (**a**) FC (−10.6 °C/s), (**b**) MC (−4.6 °C/s), (**c**) SC (−2.5 °C/s) specimens according to the tempering conditions. Tempering conditions represented in black, red, green and blue mean quenching, 450, 530 and 600 °C, respectively.

As the CR decreased, hardness under the same tempering condition decreased (Figure 8). Hardness decreased as a result of the change from LBF and AF to QPF in the matrix and from M to DP/P in the secondary phase. FC-Q, MC-Q and SC-Q had hardness values of 19, 15.7 and 11.7 HRC, respectively. Grain size in each specimen was not quantified, so the contribution of the grain size could not be calculated. However, as CR increased, the grain size decreased and the hardness increased, so we infer that the grain refinement depending on the CR also influenced the increased hardness [18,20,46].

The degree of hardness decreased after tempering and the range of the decrease increased as *T*_TEM_ and *t*_TEM_ increased. The range of reduction was decreased as the CR during quenching slowed. The hardness of FC specimens decreased by 5.2 HRC from 19 (FC-Q) to 14.8 HRC (FC-650_5); the hardness of MC specimens decreased by 4.9 HRC from 15.7 (MC-Q) to 10.8 HRC (MC-650_5), and the hardness of SC specimens decreased by 3.8 HRC from 11.7 (SC-Q) to 7.9 HRC (SC-650_5). Previous studies [2,47,48,49] have reported that *T*_TEM_ changes the hardness of M, LBF, P and ferrite and that the decreasing range of hardness is in the order of M, LBF and P and is highly dependent on *T*_TEM_. In contrast, the hardness of ferrite was not much affected by tempering.

Decomposition of the secondary phase increased as *T*_TEM_ and *t*_TEM_ increased (Figure 5 and Figure 6). Hardness was reduced by decomposition and softening of hard secondary phases that were enriched in carbon. Tempering of LBF decreased the hardness due to the diffusion of carbon in LBF and the reduction in the dislocation density. The major reasons for the change in the hardness of ferrite were reduced residual stress and reduced dislocation density [47,49]. Tempering reduced hardness by releasing residual stress, decreasing the dislocation density and causing precipitation of solute carbon in the microstructure.

**Figure 9:**
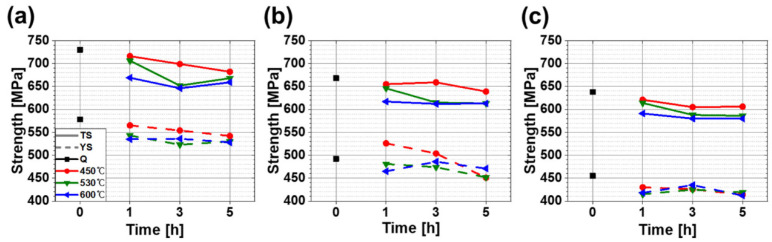
Tensile properties of (**a**) FC (−10.6 °C/s), (**b**) MC (−4.6 °C/s), (**c**) SC (−2.5 °C/s) specimens according to tempering conditions. The solid line and dotted line show tensile strength and yield strength. Tempering conditions represented in black, red, green and blue mean quenching, 450, 530 and 600 °C, respectively.

Tensile strength and yield strength decreased with decreasing CR under the same tempering conditions. The tensile strength and yield strength were highest in FC-Q (730 and 578 MPa, respectively) (Figure 9a), followed by MC-Q (668, 492 MPa) (Figure 9b) and SC-Q (638, 455 MPa) (Figure 9c). All specimens had high strength due the high content of GBF in the microstructure. Microstructure types and their fractions were affected by CR and affected the samples’ strengths (Table 3, Figure 4).

Strengths were decreased after tempering (Figure 9), but not as quickly as hardness (Figure 8). Decomposition of the secondary phase was caused by precipitation of solute carbon during tempering (Figure 6). Carbon is in solid solution and has a strong strengthening effect; therefore the strength decreased significantly as the carbon content in the secondary phase decreased due to the diffusion of carbon atoms into M_3_C cementite carbides during tempering [10]. The formation of the carbides was indeed observed in tempered specimens, as shown in Figure 7.

The reduction ranges of strength after tempering under CRs were defined as the difference between the Q specimen and the tempered specimen that had the lowest strength. In FC specimens, the reduction ranges were 80 MPa for tensile strength and 55 MPa for yield strength; in both MC and SC specimens, the reduction ranges were 60 MPa for tensile strength and 40 MPa for yield strength. Tempering reduced strength by decomposing hard phases, and the reduction ranges increased with the increase in the area fraction of the hard phase. The similarity of the reduction ranges of strength in MC and SC may be a result of complex phenomena that include not only the fraction of the hard phase but also grain size and fine precipitates.

**Figure 10:**
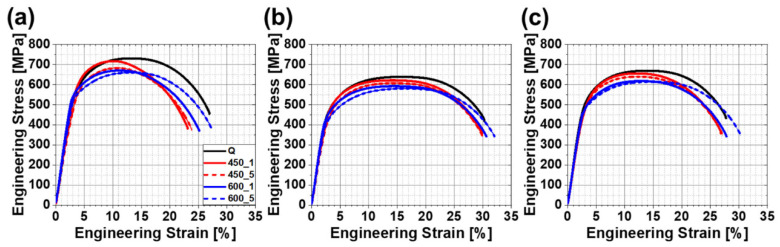
Engineering stress–strain curves of (**a**) FC (−10.6 °C/s), (**b**) MC (−4.6 °C/s), (**c**) SC (−2.5 °C/s) specimens according to the tempering conditions. Line colors and patterns distinguish tempering temperature: black is quenching, red is 450 °C and blue is 600 °C, respectively. Solid lines are 1 h, dotted lines are 5 h (excluded quenching).

Strain–stress curves of the entire specimen showed dome-shaped, continuous yielding behavior (Figure 10). This phenomenon could be related to high mobile dislocation density without pinning effect of carbons or precipitates [50,51,52,53]. On the contrary, some previous studies have shown discontinuous yielding after tempering or ageing [50,51,52,53,54,55,56]. It is well accepted that discontinuous yielding is caused by unpinning of dislocation from the Cottrell atmosphere, which formed by the interaction between dislocation and interstitial atoms such as carbon and nitrogen [52,54,55]. Heat treatment could induce diffusion of interstitial atoms into the dislocation core so dislocation is pinned during deformation and able to escape from the atmosphere by reaching the upper yield point. Moreover, this discontinuous yielding can be caused by the pinning effect of fine precipitates of critical size on dislocation [53]. For breaking away from pinning precipitates, higher stress will be required to drive dislocation. Therefore, it can be postulated that both chemical composition and the tempering conditions of this study may have been insufficient to form the Cottrell atmosphere or to grow precipitates to the critical size that is necessary for the development of discontinuous yield behavior.

**Figure 11:**
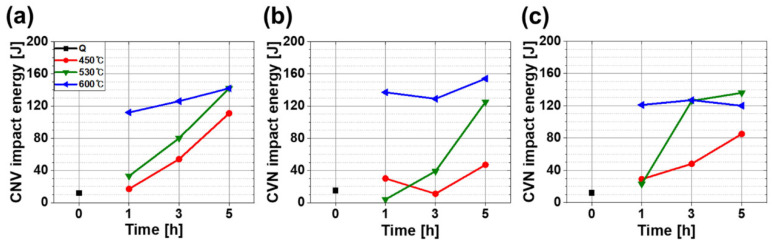
CVN impact energy of (**a**) FC (−10.6 °C/s), (**b**) MC (−4.6 °C/s), (**c**) SC (−2.5 °C/s) specimens according to the tempering conditions. Colors indicate tempering conditions: black, red, green and blue mean quenching, 450, 530 and 600 °C, respectively.

Chary V-notch (CVN) impact energy was not significantly affected by CR (Figure 11). The experimental temperature (−46 °C) was assumed to be in the ductile-to-brittle transition temperature (DBTT) range. Steels that had a similar composition to that of the steels in this experiment had DBTTs in the range of −25 °C to −100 °C, as in earlier investigations [9,16,30,57,58,59].

**Figure 12:**
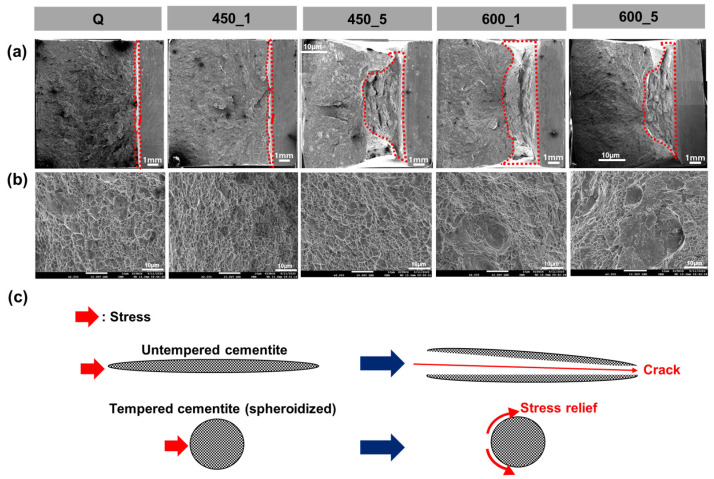
(**a**) Increased ductile-fractured surface (inside red lines) with tempering, (**b**) dimpled surface, (**c**) schematic illustration of stress relief on spheroidized carbides.

After tempering, all specimens under various CRs had increased CVN impact energy (Figure 10). It increased with both *T*_TEM_ and *t*_TEM_. By observing the fracture surface (Figure 12a,b), it was found that tempering increased the area fraction of the dimpled surface (red lines in Figure 12a), indicating that tempering promoted ductile fracture. The size distribution of the dimple did not show any feasible difference after tempering at 450 °C but became irregular after tempering at 600 °C. This increase in CVN impact energy could be possibly explained with the tempering effect which induced martensite to lose its brittleness by rejecting carbons and annihilating dislocation. Moreover, as evidenced in Figure 6, the rejected carbons formed cementite carbides in tempered martensite and those carbides were spheroidized with increasing tempering time and temperature. It can be postulated that the carbides with high aspect ratio could cause local concentrated stress, which can lead to fracture, but spheroidized cementite relieves local concentrated stress, and thereby inhibit initiation and propagation of cracks and increases toughness (Figure 12c) [13]. Tempering also reduces dislocation density and residual stress, and these changes also contribute to the increase in toughness [19,60,61,62].

## 5. Conclusions

The present work investigated the effect of heat treatment on microstructure and mechanical properties of flanges by directly obtaining local properties of interest from the flanges under the heat treatment process. Conclusions are as follows:

(1) During quenching, cooling rates varied among locations due to non-uniform cooling in flanges. Microstructure was strongly affected by cooling rates in a way that area fraction of either hard or soft constituent phases was determined by cooling rate. FC-Q has the highest area fraction of hard phase such as LBF, AF and M while SC-Q showed the area volume fraction of softer phases like QPF and DP/P.
(2) Both strength and hardness were dependent on cooling rates; faster cooling rates induced hard phases so that hardness and strength resultantly increased. CVN impact energy at −46 °C, however, did not show clear dependence on cooling rates.
(3) Tempering evidently changed microstructure by decomposing of secondary phases such as M and P and spheroidizing cementite carbide. Accordingly, hardness, strength and CVN impact energy were improved.

## Figures and Tables

**Table 1:** Chemical composition (mass %) of the present steel sheet.

Element	Fe	C	Si	Mn	P	S	Cr	Ni	As
Content	Bal.	0.1605	0.246	1.266	0.0147	0.0045	0.191	0.014	0.004
**B**	**Ca**	**Cu**	**Mo**	**N**	**Nb**	**Sn**	**Ti**	**V**	**Al**
0.0002	0.0002	0.027	0.082	0.0037	0.0019	0.002	0.0016	0.054	0.026

**Table 2:** Post-heat treatment conditions and symbols.

Specimen Name	Tempering Temperature (°C)	Tempering Time (h)
Q	×	×
450_1	450	1
450_3	450	3
450_5	450	5
530_1	530	1
530_3	530	3
530_5	530	5
600_1	600	1
600_3	600	3
600_5	600	5

**Table 3:** Microstructure and area fraction (%) of experimental steel according to the CR in the quenching-only treatment, Q.

Name	Matrix				Secondary Phase		
	LBF	AF	GBF	QPF	M	DP/P	MA
FC-Q	13 ± 1.0	16 ± 4.0	47 ± 4.1	-	17 ± 2.0	5 ± 1.5	2 ± 0.2
MC-Q	8 ± 3.0	12 ± 4.4	51 ± 4.7	6 ± 3.4	11 ± 3.1	10 ± 0.7	2 ± 0.1
SC-Q	-	-	37 ± 7.9	39 ± 5.9	7 ± 1.3	15 ± 1.4	2 ± 0.1
